# Clinical risk score for early prediction of recurring SARS-CoV-2 positivity in non-critical patients

**DOI:** 10.3389/fmed.2022.1002188

**Published:** 2023-02-01

**Authors:** Anni Li, Chao Wang, An Cui, Lingyu Zhou, Wei Hu, Senlin Ma, Dian Zhang, Hong Huang, Mingquan Chen

**Affiliations:** ^1^Shanghai Key Laboratory of Infectious Diseases and Biosafety Emergency Response, Department of Infectious Diseases, National Medical Center for Infectious Diseases, Huashan Hospital, Fudan University, Shanghai, China; ^2^Department of Emergency Medicine, Huashan Hospital, Fudan University, Shanghai, China; ^3^Information Center, Huashan Hospital, Fudan University, Shanghai, China

**Keywords:** risk score, SARS-CoV-2, non-critical, recurring, COVID-19, re-positive

## Abstract

**Introduction:**

Recurrent positive results in quantitative reverse transcriptase-PCR (qRT-PCR) tests have been commonly observed in COVID-19 patients. We aimed to construct and validate a reliable risk stratification tool for early predictions of non-critical COVID-19 survivors’ risk of getting tested re-positive within 30 days.

**Methods:**

We enrolled and retrospectively analyzed the demographic data and clinical characters of 23,145 laboratory-confirmed cases with non-critical COVID-19. Participants were followed for 30 days and randomly allocated to either a training (60%) or a validation (40%) cohort. Multivariate logistic regression models were employed to identify possible risk factors with the SARS-CoV-2 recurrent positivity and then incorporated into the nomogram.

**Results:**

The study showed that the overall proportion of re-positive cases within 30 days of the last negative test was 24.1%. In the training cohort, significantly contributing variables associated with the 30-day re-positivity were clinical type, COVID-19 vaccination status, myalgia, headache, admission time, and first negative conversion, which were integrated to build a nomogram and subsequently translate these scores into an online publicly available risk calculator (https://anananan1.shinyapps.io/DynNomapp2/). The AUC in the training cohort was 0.719 [95% confidence interval (CI), 0.712–0.727] with a sensitivity of 66.52% (95% CI, 65.73–67.30) and a specificity of 67.74% (95% CI, 66.97–68.52). A significant AUC of 0.716 (95% CI, 0.706–0.725) was obtained for the validation cohort with a sensitivity of 62.29% (95% CI, 61.30–63.28) and a specificity of 71.26% (95% CI, 70.34–72.18). The calibration curve exhibited a good coherence between the actual observation and predicted outcomes.

**Conclusion:**

The risk model can help identify and take proper management in high-risk individuals toward the containment of the pandemic in the community.

## Highlights

- Question: Whether a reliable risk stratification tool composed of epidemiological and clinical characteristics can be conducted and utilized to predict probability of retesting positivity within 30 days for non-critical patients?
- Findings: In this study of 23,145 SARS-CoV-2 infection cases, a multivariate model presented with a nomogram and a web-based risk calculator were developed and validated to predict the probability of retesting as SARS-CoV-2 RNA positive within 30 days among non-critical patients who had got negative result(s). The risk score included six values: clinical type, COVID-19 vaccination status, myalgia, headache, admission time, and first negative conversion.
- Meaning: This model-derived risk score can be used to help identify and manage high-risk individuals early for containing the spread of the pandemic in the community.

## Introduction

Recently emerging coronavirus disease-2019 (COVID-19) has created a major public health challenge globally. A highly contagious severe acute respiratory syndrome coronavirus 2 (SARS-CoV-2) strain is identified as the primary cause of COVID-19 symptoms. As of 24 May, 2022, the COVID-19 pandemic has been lingering in most countries, accounting for 161,523,786,368 confirmed cases and claiming 6,279,667 lives, according to the World Health Organization (WHO) data (1).

Currently, quantitative reverse transcriptase-PCR (qRT-PCR)-based screening and detection of SARS-CoV-2 infection is one of the standard laboratory diagnostic methods. Recurrent positive results in qRT-PCR tests have been commonly observed in COVID-19 patients. Not only that, patients could be also alternatively tested as negative and positive or even re-positive after confirmed double negative tests, as commonly noticed in many patients who got discharged from hospitals after recovery (2–5).

Recently, the Omicron variant has rapidly replaced the Delta as the most dominant one in many geographical regions owing to its highest infectivity, transmissibility, and immune evasion capabilities (6). Most COVID-19 patients infected with the Omicron were either asymptomatic or presented mild symptoms and were released from quarantine within a few days. It is still puzzling whether these patients are contagious when they get re-tested as positive for SARS-CoV-2. For emerging infectious diseases like COVID-19, it is critical to understand the way to gauge the viral shedding kinetics and identify different epidemiological parameters to predict the cross-infection capacity of COVID-19 patients who tested re-positive. Here, we aimed to retrospectively examine the clinical features of SARS-CoV-2 RNA re-emergence among non-critical cases and develop a risk tool to assess re-positivity at the early stage as well as to conduct tests for negative-positive patients.

## Materials and methods

### Study design and participants

This study was designed to examine factors related to recurrent PCR positivity for SARS-CoV-2 viral RNA and develop a risk prediction platform for patients diagnosed with non-critical COVID-19. Between 5 April, 2022 and 19 April, 2022, data on laboratory-confirmed COVID-19 cases were consecutively accumulated from Fangcang Shelter Hospital, which was a temporary designated hospital serving to isolate non-critical COVID patients. These patients were followed up for 30 days from the date of admission and underwent a minimum of three rounds of qRT-PCR tests for SARS-CoV-2 infection with at least a 24-h interval between any two consecutive assays. The cases were excluded if: (1) the patient’s record did not contain essential information; (2) the patient was readmitted; (3) the patient was classified as having at least one of the following critical conditions like mechanical ventilation due to respiratory distress, shock syndromes, or intensive care support for multi-organ failure; (4) follow-ups were not maintained; (5) missed out three RT-PCR tests; and (6) had persistently positive results on RT-PCR tests. The diagnosis and clinical classification of COVID-19 were conducted following the ninth edition of the Chinese Clinical Guidance for COVID-19 pneumonia diagnosis and treatment. The enrolled cases were considered confirmed if the RT-PCR test from pharyngeal or nasal swabs revealed positive results for SARS-CoV-2 infection.

### Data collection

The clinical data of COVID-19 patients were collected by experienced clinicians and subsequently cross-verified, which included demographics, clinical symptoms and signs, relevant comorbidities, vaccination status, and RT-PCR test results. Demographic variables were age, sex, and admission time (the time difference between the day of the first positive test and hospitalization). Clinical symptoms and signs included cough, excessive sputum production, fatigue, myalgia, fever, sore throat, runny nose, hypogeusia, headache, hyposmia, nasal congestion, chest distress, and diarrhea. Comorbidities on admission were self-reported, which included diabetes, hypertension, chronic pulmonary disease, hepatitis B, cancer, and asthma. Reported vaccinations were BBIBP-CorV, Ad5-nCoV2, and CoronaVac, according to the information administration system. Vaccination status was categorized either as partially vaccinated if received one dose; primarily vaccinated if taken two doses; completely vaccinated if received a booster dose in addition to complete primary vaccination; or unvaccinated. Detection results were divided into positive and negative groups, respectively, depending on whether the cycle threshold (Ct) value was < 35 or > 35, reflecting the viral load. On admission, patients were allocated to the medium-high (Ct ≤ 30) and low (Ct > 30) viral load groups based on their first RT-PCR reports. The first negative conversion duration (days) was defined as the number of days between the first positive and the first negative result. Viral shedding duration referred to the number of days from the first positive to the first of two consecutive negative RT-PCR tests. The result was considered negative if the results of two consecutive RT-PCR tests were negative. Admission time was the duration of time between the first positive result and admission to the hospital. Recurrence frequency and the number of negative RT-PCR results before recurrence were respectively determined based on the frequency of positive tests after the negative test, and the frequency of negative tests before the positive test within 30 days.

### Statistical analysis

The data were tested for a normal distribution. Continuous variables were presented as the mean ± standard deviation (x ± SD) or interquartile range (IQR) and median, as appropriate. Comparisons between groups were performed either by *t*-test for normally distributed or Mann–Whitney U test for irregularly distributed continuous variables. Categorical variables were presented as frequencies (*n*) and percentages (%). The Fisher’s exact test or Chi-square test was performed to analyze the significance. In the training cohort, a univariate logistic regression model was applied to assess the significance of each variable as the independent risk factor for the recurrent COVID-19 positive results. Stepwise logistic regression analysis was used for multivariate analysis identify independent risk factors for recurrence. The inclusion criteria were *p* ≤ 0.05, and the exclusion criteria was *p* ≥ 1.0. Subsequently, a nomogram and related web calculator analysis were conducted using the multivariate analysis results. The receiver operating characteristic (ROC) curve, area under the ROC (AUROC), and calibration plots were used to discriminate the model performances of the validation cohort from the training cohort. The clinical impact curve (CICA), and decision curve (DCA) analyses were carried out to evaluate the clinical utility of the nomogram. The sensitivity, predictive capacity, specificity, and likelihood were considered in assessing the accuracy of the optimal cutoff. Statistical analyses were done by SPSS v26.0 (SPSS Inc., USA), SAS v9.3 (SAS Institute Inc.), and R v3.0^1^. *P*-value of < 0.05 was taken into consideration for significance analysis.

## Results

### Patients’ characteristics

The case selection flow is illustrated in Supplementary Figure 1. As of 22nd May, 2022, 25,158 patients were consecutively admitted to the hospital with the RT-PCR–confirmed SARS-CoV-2 infections. Finally, 23,145 patients were eligible to be enrolled for analysis, involving 13,887 (60%) participants in the training and 9,258 (40%) patients in the validation cohorts (Table 1). The median age was 35 (range, 1–83) years; 14,991 (64.77%) subjects were male, and 19,972 (86.29%) patients were asymptomatic carriers. SARS-CoV-2-related symptomatology was retrieved for 4,972 cases. The most common symptoms were cough [4,607 (19.9%)], and excessive sputum production [2,907 (12.56%)], followed by fatigue [1,281 (5.53%)], myalgia [1,019 (4.4%)], and fever [829 (3.58%)]. Overall, 12,219 (52.79%) patients were completely vaccinated, where CoronaVac recipients accounted for 67.56%, and 2,728 (11.79%) individuals were unvaccinated. Hypertension [2,003 (8.65%)], diabetes [641 (2.77%)], and hepatitis B infection [101 (0.44%)] were the top three comorbidities. Admissions occurred in the median of 3 (range, 2–5) days. The median length of the first negative conversion was 5 (range, 4–7) days, and the median viral shedding time was 6 (range, 4–8) days. The number of patients having at least one positive re-test within 30 days of the first positive test was 5,578 (24.1%), including 3,393 of 13,887 patients (24.43%) from training and 2,185 of 9,258 patients (23.60%) from the validation cohorts. Of these, 80.20% of cases had Ct > 30, while the rest showed Ct ≤ 30. Furthermore, 4,484 (19.37%), 938 (4.05%), and 156 (0.67%) patients reportedly had one, two, and more recurrences, respectively; 4,649 (20.09%), 889 (3.84%), and 40 (0.17%) patients were tested positive after testing negative once, twice, and thrice, respectively. There were no remarkable differences in the baseline clinical values between the validation and training cohorts.

**TABLE 1:** Baseline characteristics of the study cohort.

Characteristic	All patients (*n* = 23,145)	Training cohort (*n* = 13,887)	Validation cohort (*n* = 9,258)	*P*-value
Gender				0.472
Male	14,991 (64.77)	8,969 (64.59)	6,022 (65.05)	
Female	8,154 (35.23)	4,918 (35.41)	3,236 (34.95)	
Age, median (IQR), years	35.00 (26.00–57.00)	35.00 (26.00–57.00)	35.00 (26.00–57.00)	0.200
Age distribution				0.496
0–6 years	81 (0.35)	45 (0.32)	36 (0.39)	
7–18 years	739 (3.19)	423 (3.05)	316 (3.41)	
19–59 years	20,479 (88.48)	12,301 (88.58)	8,178 (88.33)	
60–79 years	1,820 (7.86)	1,103 (7.94)	717 (7.74)	
≥ 80 years	26 (0.11)	15 (0.11)	11 (0.12)	
Clinical type^a^				0.776
Critical	0 (0)	0 (0)	0 (0)	
Severe	3 (0.01)	1 (0.01)	2 (0.02)	
General	139 (0.6)	84 (0.6)	55 (0.59)	
Mild	3,031 (13.1)	1,830 (13.18)	1,201 (12.97)	
Asymptomatic	19,972 (86.29)	11,972 (86.21)	8,000 (86.41)	
COVID-19 vaccination status^b^				0.005
Unvaccinated	2,728 (11.79)	1,700 (12.24)	1,028 (11.10)	
Partially vaccinated	850 (3.67)	518 (3.73)	332 (3.59)	
Primarily vaccinated	7,348 (31.75)	4,304 (30.99)	3,044 (32.88)	
Completely vaccinated	12,219 (52.79)	7,365 (53.04)	4,854 (52.43)	
Vaccine type				
BBIBP-CorV	7,192 (31.07)	4,334 (31.21)	2,858 (30.87)	0.586
Ad5-nCoV2	189 (0.82)	111 (0.80)	78 (0.84)	0.720
CoronaVac	15,636 (67.56)	9,367 (67.45)	6,269 (67.71)	0.676
**Sign and symptom^c^**				
Cough	4,607 (19.90)	2,775 (19.98)	1,832 (19.79)	0.720
Sputum production	2,907 (12.56)	1,764 (12.70)	1,143 (12.35)	0.423
Fatigue	1,281 (5.53)	757 (5.45)	524 (5.66)	0.496
Myalgia	1,019 (4.40)	607 (4.37)	412 (4.45)	0.774
Fever	829 (3.58)	468 (3.37)	361 (3.90)	0.034
Sore throat	193 (0.83)	119 (0.86)	74 (0.80)	0.637
Running nose	150 (0.65)	94 (0.68)	56 (0.60)	0.504
Headache	69 (0.30)	37 (0.27)	32 (0.35)	0.279
Hypogeusia	10 (0.04)	8 (0.06)	2 (0.02)	0.197
Hyposmia	18 (0.08)	10 (0.07)	8 (0.09)	0.700
Nasal congestion	155 (0.67)	89 (0.64)	66 (0.71)	0.511
Chest distress	47 (0.20)	26 (0.19)	21 (0.23)	0.512
Diarrhea	34 (0.15)	12 (0.09)	22 (0.24)	0.003
Comorbidities^d^				
Hypertension	2,003 (8.65)	1,201 (8.65)	802 (8.66)	0.970
Diabetes	641 (2.77)	364 (2.62)	277 (2.99)	0.092
Cancer	38 (0.16)	30 (0.22)	8 (0.09)	0.017
Hepatitis B infection	101 (0.44)	62 (0.45)	39 (0.42)	0.776
Chronic pulmonary disease	88 (0.38)	52 (0.37)	36 (0.39)	0.862
Asthma	37 (0.16)	26 (0.19)	11 (0.12)	0.202
History of allergies	573 (2.48)	353 (2.54)	220 (2.38)	0.427
Admission time,^e^ median (IQR), days	3 (2–5)	3 (2–5)	3 (2–5)	0.287
First negative conversion,^f^ median (IQR), days	5 (4–7)	5 (4–7)	5 (4–7)	0.838
Viral shedding time,^g^ median (IQR), days	6 (4–8)	6 (48)	6 (4–8)	0.689
Viral nucleic acid Ct value group				0.716
Value > 30	18,563 (80.20)	11,127 (80.13)	7,436 (80.32)	
Value ≤ 30	4,582 (19.80)	2,760 (19.87)	1,822 (19.68)	
Recurrence				0.147
Non-recurrent group	17,567 (75.90)	10,494 (75.57)	7,073 (76.40)	
Recurrent group	5,578 (24.10)	3,393 (24.43)	2,185 (23.60)	
Recurrence frequency				0.096
0	17,567 (75.90)	10,494 (75.57)	7,073 (76.40)	
1	4,484 (19.37)	2,698 (19.43)	1,786 (19.29)	
2	938 (4.05)	594 (4.28)	344 (3.72)	
3	156 (0.67)	101 (0.73)	55 (0.59)	
Number of negative PCR before recurrence				0.069
0	17,567 (75.90)	10,494 (75.57)	7,073 (76.40)	
1	4,649 (20.09)	2,830 (20.38)	1,819 (19.65)	
2	889 (3.84)	532 (3.83)	357 (3.86)	
3	40 (0.17)	31 (0.22)	9 (0.10)	

IQR, interquartile range. ^a^Patients were categorized according to the 9th edition of clinical guidance for COVID-19 pneumonia diagnosis and treatment of China. ^b^Vaccination status was assigned at the time of admission. ^c^Self-reported by participants, and categorized by investigators following federal government standards; percentages do not sum to 100% by column.

^d^Self-reported by participants; percentages do not sum to 100% by column. ^e^Indicates the interval between the first positive SARS-CoV-2 RNA test and the date of the clinical interview.

^f^The interval between the first positive and the first negative results. ^g^Referred to the duration from the first positive to the first of two consecutive negative RT-PCR tests.

### Predictor selection

The univariate logistic analysis result for the training cohort is presented in Table 2. Eleven variables, including gender, age without distribution, clinical type, cough, fever, headache, fatigue, myalgia, admission time, first negative conversion time, and Ct value cutoff, were identified as significant predictors of COVID-19 recurrence. The prediction model using multivariate logistic regression indicated six variables that were independent predictors of SARS-CoV-2 re-positivity and included in the risk score (Table 3). These variables were clinical type [odds ratio, OR: 0.87; 95% confidence interval (CI): 0.79–0.97; *P* = 0.014], COVID-19 vaccination status (OR: 0.96; 95% CI: 0.92–0.99; *P* = 0.024), myalgia (OR: 1.31; 95% CI: 1.09–1.58; *P* = 0.005), headache (OR: 2.10; 95% CI: 1.06–4.18; *P* = 0.034), admission time (OR: 0.58; 95% CI: 0.56–0.60; *P* < 0.001), and first negative conversion (OR: 1.47; 95% CI: 1.43–1.51; *P* < 0.001).

**TABLE 2:** Demographics and characteristics of patients infected with SARS-CoV-2 in the training cohort.

Characteristic	Non-recurrent positive (*n* = 10,494)	Recurrent positive (*n* = 3,393)	*P*-value
Gender			0.037
Male	6,727 (64.10)	2,242 (66.08)	
Female	3,767 (35.90)	1,151 (33.92)	
Age, median (IQR), years	35.00 (26.00–57.00)	35.00 (26.00–56.00)	<0.001
Age distribution			0.123
0–6 years	37 (0.35)	8 (0.24)	
7–18 years	341 (3.25)	82 (2.42)	
19–59 years	9,270 (88.34)	3,031 (89.33)	
60–79 years	835 (7.96)	268 (7.90)	
≥ 80 years	11 (0.10)	4 (0.12)	
Clinical type			<0.001
Critical	0 (0.00)	0 (0.00)	
Serious	0 (0.00)	1 (0.03)	
General	61 (0.58)	23 (0.68)	
Mild	1,314 (12.52)	516 (15.21)	
Asymptomatic	9,119 (86.90)	2,853 (84.08)	
COVID-19 vaccination status			0.093
Unvaccinated	1,251 (11.92)	449 (13.23)	
Partially vaccinated	380 (3.62)	138 (4.07)	
Primarily vaccinated	3,285 (31.30)	1,019 (30.03)	
Completely vaccinated	5,578 (53.15)	1,787 (52.67)	
Vaccine type			
BBIBP-CorV	3,277 (31.23)	1,057 (31.15)	0.935
Ad5-nCoV2	85 (0.81)	26 (0.77)	0.804
CoronaVac	7,068 (67.35)	2,299 (67.76)	0.662
**Sign and symptom**			
Cough	2,049 (19.53)	726 (21.4)	0.018
Sputum production	1,341 (12.78)	423 (12.47)	0.635
Fever	324 (3.09)	144 (4.24)	0.001
Sore throat	88 (0.84)	31 (0.91)	0.680
Hypogeusia	6 (0.06)	2 (0.06)	0.970
Hyposmia	8 (0.08)	2 (0.06)	0.744
Headache	22 (0.21)	15 (0.44)	0.022
Running nose	64 (0.61)	30 (0.88)	0.090
Fatigue	531 (5.06)	226 (6.66)	<0.001
Myalgia	407 (3.88)	200 (5.89)	<0.001
Nasal congestion	72 (0.69)	17 (0.50)	0.240
Chest distress	20 (0.19)	6 (0.18)	0.872
Diarrhea	9 (0.09)	3 (0.09)	0.964
Comorbidities			
Hypertension	919 (8.76)	282 (8.31)	0.422
Diabetes	266 (2.53)	98 (2.89)	0.263
Cancer	21 (0.20)	9 (0.27)	0.477
Hepatitis B infection	49 (0.47)	13 (0.38)	0.525
Chronic pulmonary disease	41 (0.39)	11 (0.32)	0.581
Asthma	19 (0.18)	7 (0.21)	0.767
History of allergies	263 (2.51)	90 (2.65)	0.638
Admission time, median (IQR), days	4 (2–6)	2 (1–4)	<0.001
First negative conversion, median (IQR), days	5 (4–7)	5 (4–7)	<0.001
Viral nucleic acid Ct value group			<0.001
Value > 30	8,506 (81.06)	2,621 (77.25)	
Value ≤ 30	1,988 (18.94)	772 (22.75)	

IQR, interquartile range.

**TABLE 3:** Multivariate logistic regression analysis of recurrent positive based on data in the training cohort.

Variable	β	OR (95% CI)	*P*
Clinical type	−0.134	0.87 (0.79–0.97)	0.014
COVID-19 vaccination status	−0.046	0.96 (0.92–0.99)	0.024
Myalgia	0.270	1.31 (1.09–1.58)	0.005
Headache	0.744	2.10 (1.06–4.18)	0.034
Admission time	−0.546	0.58 (0.56–0.60)	<0.001
First negative conversion	0.386	1.47 (1.43–1.51)	<0.001

OR, odds ratio.

### Construction and validation of the nomogram

**FIGURE 1:**
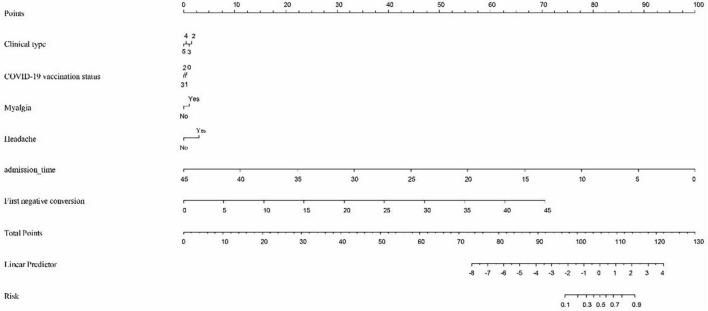
To calculate predicted occurrence, locate an individual’s value on each independent variable axis, and then draw a straight line upward “Points” row to obtain the points for each variable. Next, locate the sum of these points on the total points axis, and draw a line downward to the “Risk” row to obtain the probability of re-testing positivity within 30 days. Clinical type: 2—Severe, 3—General, 4—Mild, and 5—Asymptomatic. COVID-19 vaccine status: 0—Unvaccinated, 1—Partially vaccinated, 2—Primarily vaccinated, and 3—Completely vaccinated.

**FIGURE 2:**
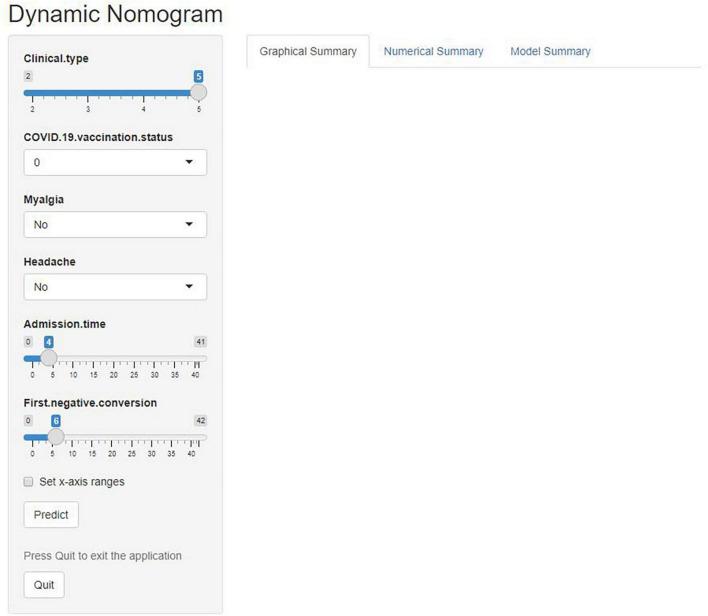
An online calculator was developed to automatically calculate the risk of re-testing positivity among non-critical individuals within 30 days by entering the values of the six variables. Clinical type: 2—Severe, 3—General, 4—Mild, and 5—Asymptomatic. COVID-19 vaccine status: 0—Unvaccinated, 1—Partially vaccinated, 2—Primarily vaccinated, and 3—Completely vaccinated.

**FIGURE 3:**
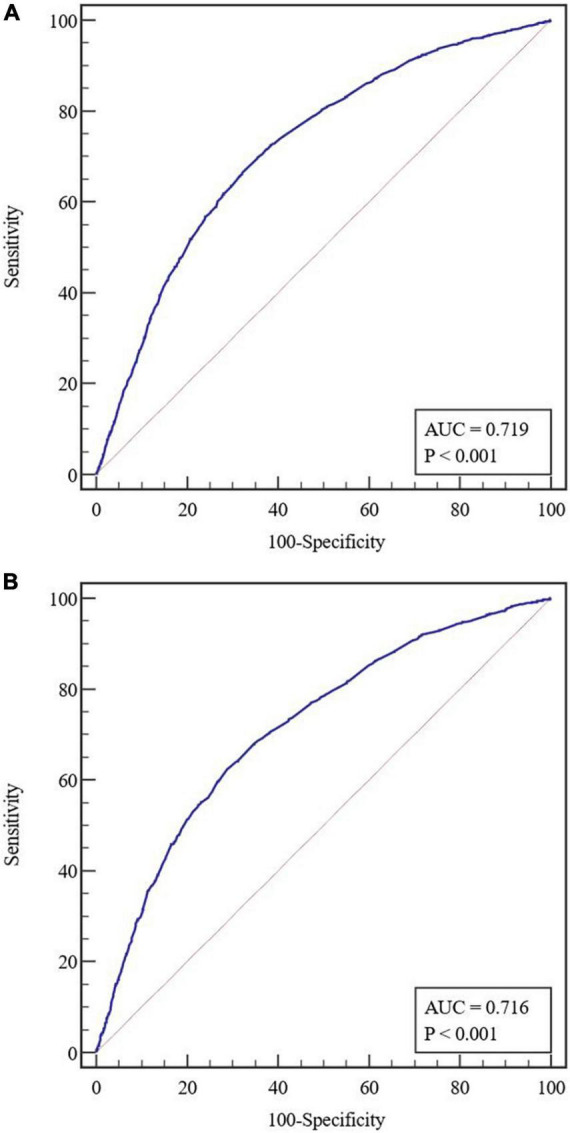
**(A)** Receiver operating characteristic (ROC) curves in the training cohort. **(B)** Receiver operating characteristic curves in the validation cohort. AUC, area under the receiver operating characteristic curve.

**FIGURE 4:**
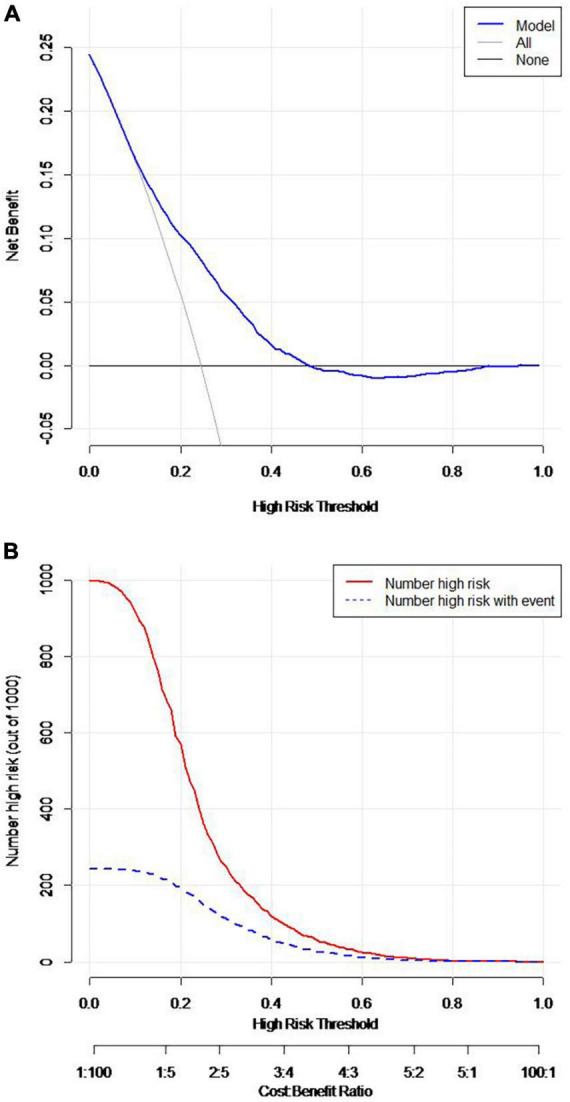
**(A)** Decision curve (DCA). **(B)** Clinical impact curve (CICA).

The predictive nomogram was composed of the aforementioned six clinical features for predicting the incidence rate of COVID-19 positive re-test in the training cohort (Figure 1). An online calculator was developed to allow subjects to estimate their risk scores by entering values of pre-determined six clinical features with the likelihood that a less severe patient would present at least one re-positive result after negative tests^2^ (Figure 2). The AUROC value for predicting a positive re-test within 30 days showed the good discriminative ability of the model in both the training (0.719, 95% CI: 0.712–0.727), and validation (0.716, 95% CI: 0.706–0.725) cohorts (Figures 3A, B). The calibration curves for the nomogram-predicted and actual observations were consistent between the groups with bootstrap (Supplementary Figures 2A, B). DCA and CICA assessed the clinical utility of the nomogram, which showed the superior net benefit of threshold probabilities within the wide and practical ranges (Figures 4A, B).

### Risk of 30-day positive re-testing based on the predictive nomogram

The cutoff value of the nomogram was 101. The sensitivity, positive prediction, specificity, and negative prediction values were 66.52, 40.00, 67.74, and 86.22% in the training cohort; and 62.29, 40.10, 71.26, and 85.95% in the validation cohort, respectively, for predicting the 30-day positive re-testing for COVID-19 (Table 4).

**TABLE 4:** Accuracy of the prediction score of the nomogram for estimating the risk of recurrent positive.

Variable	Value (95% CI)	
	Training cohort	Validation cohort
Area under ROC curve,	0.719 (0.712–0.727)	0.716 (0.706–0.725)
Cutoff score	101	101
Sensitivity, %	66.52 (65.73–67.30)	62.29 (61.30–63.28)
Specificity, %	67.74 (66.97–68.52)	71.26 (70.34–72.18)
Positive predictive value, %	40.00 (39.19–40.82)	40.10 (39.10–41.10)
Negative predictive value, %	86.22 (85.65–86.80)	85.95 (85.24–86.66)
Positive likelihood ratio	2.062 (2.026–2.099)	2.167 (2.116–2.219)
Negative likelihood ratio	0.494 (0.486–0.503)	0.529 (0.519–0.539)

ROC, receiver operating characteristic.

## Discussion

Early prediction of positive re-testing in non-critical COVID-19 patients would be beneficial in making better management and screening policies. Here, we summarized the clinical features and viral shedding kinetics of patients with recurring SARS-CoV-2 positivity. Moreover, we identified that the milder clinical types, incomplete vaccination, myalgia, headache, reduction in admission time (might be associated with symptoms, Supplementary Table 1), and long duration of the first negative conversion were correlated with higher odds of positive re-test for COVID-19. We also established a clinical risk nomogram model as well as a web-based calculator with sufficient sensitivity and specificity to predict the probability of COVID-19 recurrence within 30 days in non-critical cases who had confirmed negative result(s). These values are generally available in any isolation sites. DCA and CICA results further supported the clinical benefit of these models.

According to the Shanghai Municipal Health Commission, as of 4th May, 2022, approximately 90% (547, 056) of 601, 942 infected cases were asymptomatic carriers, (7) which were consistent with our results (86.29% for asymptomatic). It is shown that the median duration of the virial shedding for symptomatic Omicron infected subjects is 6 (range 4–8) days (8). Notably, our cohort of mostly asymptomatic subjects showed similar virial shedding kinetics, suggesting that it might be independent of symptom presentation. Consistently, our results indicate that more severe COVID-19 patients might be at higher risk for re-positive tests (9). Interestingly, we also found that patients with a long duration of first negative conversion might be at higher risk of COVID-19 recurrence. Therefore, we hypothesized that the long duration of first negative conversion and less severe COVID-19 symptoms, especially asymptomatic ones, might elicit a weaker immune response and minimum viral clearance.

Our study presented a total of 4,649 (20.09%) individuals who had at least one negative test which turned into a positive at later stages. Initially, 889 (3.84%) patients were released from quarantine after having two consecutive negative tests (tested at 24 h intervals), but they turned positive later. Several possibilities for the positive re-tests for COVID-19 have been proposed (10–12). In the present scenario, it’s been challenging to differentiate between COVID-19 recurrence due to the delayed shedding of non-viable viral remnants and truly contagious viral particles. The patients who meet the criteria for release from quarantine still need to be monitored for symptomatic manifestations *via* RT-PCR test.

The Ct value for viral load is applied to evaluate the course of infection and virulence, with every ∼3.3 cycle increase in the Ct value reflecting a 10-fold reduction in starting material (13). Recently, any qRT-PCR values less than a cutoff Ct < 35 indicate infectious status. However, the range of Ct values (Ct: 24–35) in predicting viral infectivity varies greatly across studies (14, 15). It is found that the mean Ct for the contagious patient is very close to that of a non-infectious individual (23.99 and 24.02, respectively) (16). It has also been revealed that the average Ct for Omicron infection is higher than that of the Delta variant (17). In our research, 80.20% of patients featured a higher Ct value in the current Omicron pandemic. It might need more observations on the Ct value to confirm if a cutoff value of 35 could be universally utilized to identify individuals who are positive and/or at a higher risk of transmission.

The guidelines for isolation, admission, and discharge for SARS-CoV-2 infected patients are not universal. As per the WHO clinical management guidelines, a COVID-19 patient should be declared clinically recovered after having at least two sequential negative PCR tests (at intervals of > 24 h) at the time of discharge. The proportion of recurring COVID-19 patients varies between 2.4 and 69.2% and could occur any time between 1 and 38 days of hospital discharge (2–5). The CDC considers there should be no infectious viral particle/remnant produced in 88 and 95% of patient samples, respectively, collected at 10 and 15 days after symptom onset. Therefore, adults with asymptomatic, mildly symptomatic, moderate, and severe COVID-19 patients could be released from isolation in 5–10 days post-infection (18).

However, these observations are mainly based on the initial epidemiology of COVID-19. Omicron cases demonstrated a lot of variabilities compared with other strains, such as release from quarantine as early as < 5–10 days. Notably, the detection of viral RNA in patients does not confirm the infectious status of the subject. Hence, challenges and uncertainties still exist in the public health policies for global epidemic preparedness based on existing evidence. Although a single negative test would not be a confirmatory result for the actual viral shedding, it may suggest a connection between individualized immunity and recurrent infection. It is crucial to conduct epidemiological surveys on individuals, especially those with recurrent COVID-19 diagnosis and high viral loads, to evaluate their contagiousness and monitor health conditions.

At least seven major limitations for the findings are as follows. First, the detection frequency was varying and inconsistent after discharging from the hospital, which might underestimate the proportion of re-testing positive after discharge. Second, the data for the nomogram model were entirely obtained from the Fangcang Shelter Hospital in Shanghai, China. These patients might have had a milder illness or presented asymptomatic nature with fewer comorbid and immunodeficient conditions, which could introduce potential bias and limit the universal application of the risk model in other geographical regions. Third, other potential values (such as treatment) might affect viral shedding and were not available due to incomplete clinical data. Fourth, given that false negative results of COVID-19 PCR were an objective limitation, and PCR tests could not reflect the true viral load, virus culturing is required to determine the infectivity. Fifth, there could be bias in self-reported symptoms and comorbidities. Sixth, the prediction tool incorporates the clinical features and Immunization, despite more accessible and suitable during Omicron predominance, lack of the weight of serology, imaging, or other potential factors. Finally, although the validation cohort was conducted by external validation, the strength of the verification with retrospective cohort may not be as good as the prospective cohort.

## Conclusion

We established a risk model based on the clinical features of COVID-19 to predict the probability of re-testing positive within 30 days of negative test in less severely affected patients who were confirmed negative for SARS-CoV-2 infection in the current outbreak in Shanghai. Our model demonstrated satisfactory performance and might have clinical utilization in the early identification and management of concerned individuals.

## Data availability statement

The raw data supporting the conclusions of this article will be made available by the authors, without undue reservation.

## Ethics statement

The studies involving human participants were reviewed and approved by the Ethics Committee of the Huashan Hospital Affiliated with Fudan University. Written informed consent for participation was not required for this study in accordance with the national legislation and the institutional requirements.

## Author contributions

MC and AL designed the study. HH had full access to all of the data and took responsibility for the accuracy of data in the study. AL, LZ, and AC took responsibility for the accuracy of the data analysis and interpretation. AL drafted of the manuscript. CW, WH, SM, and DZ revised the manuscript for important intellectual content. All authors contributed to the article and approved the submitted version.

## Footnotes

1
http://www.r-project.org/

2
https://anananan1.shinyapps.io/DynNomapp2/
