# Supplementary material for: Clinical risk score for early prediction of recurring SARS-CoV-2 positivity in non-critical patients
Source: Front Med (Lausanne). 2023 Feb 1;9:1002188. doi: 10.3389/fmed.2022.1002188 (PMC9929941; doi:10.3389/fmed.2022.1002188)
Supplement: Supplementary file 1 [file Data_Sheet_1.pdf]

Supplementary Material

Supplementary Figure 1. Flow Chart of the Study

Supplementary Figure 2. Calibration curve in two cohorts

Supplementary Table 1. Subgroup analyses based on the admission time

Supplementary Figure 1. Flow diagram. The chart shows patients inclusion and exclusion in the study.

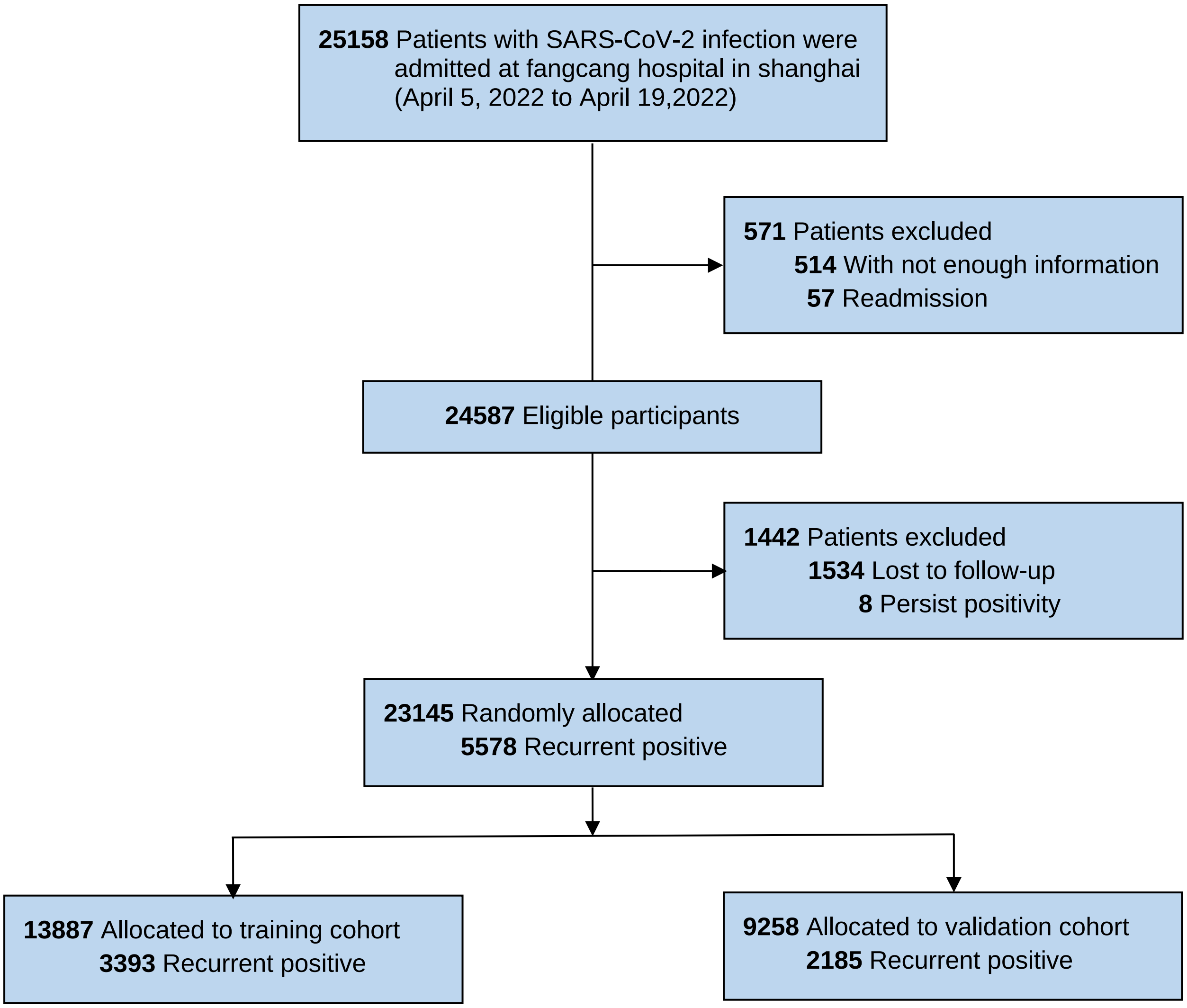

Supplementary Figure 2. Calibration curve in two cohorts

A. Calibration Curve of the Training Cohort

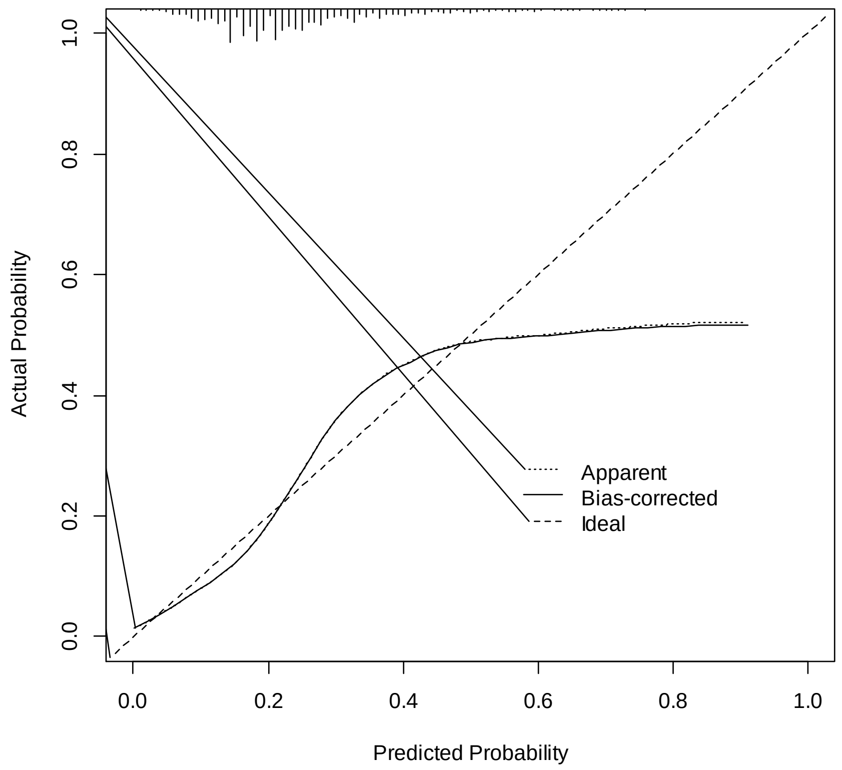

B. Calibration Curve of the Validation Cohort

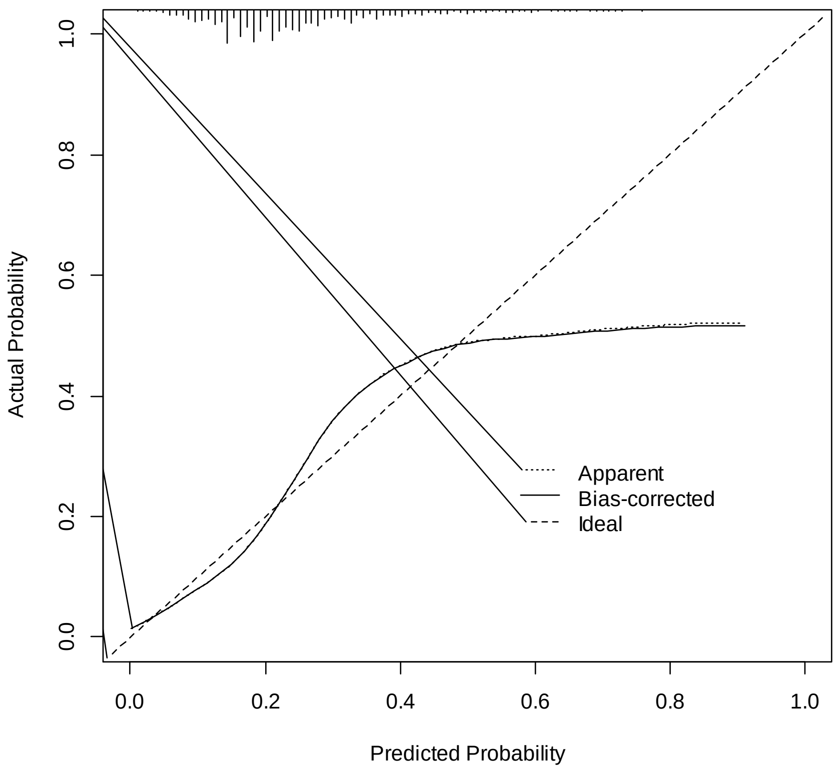

Supplementary Table 1. Subgroup analyses based on the admission time

Characteristic	Admission time≤3 (n=12500)	Admission time >3 (n=10645)	P-value
**Signs and symptoms**			0.008
Non-obvious clinical symptoms	9265(74.12)	8052(75.64)	
Marked symptoms	3235(25.88)	2593(24.36)	
Fever	511(4.09)	318(2.99)	<.001
Sore throat	132(1.06)	61(0.57)	<.001
Running nose	103(0.82)	47(0.44)	<.001
Fatigue	747(5.98)	534(5.02)	0.001
Myalgia	631(5.05)	388(3.64)	<.001
**Clinical type, n (%)**			<.001
critical	0(0)	0(0)	
serious	2(0.02)	1(0.01)	
general	67(0.54)	72(0.68)	
mild	1745(13.96)	1286(12.08)	
asymptomatic	10686(85.49)	9286(87.23)	
